# Open vs. closed reduction with pinning for displaced Rockwood and Wilkins' type C thumb metacarpal base fractures in children

**DOI:** 10.3389/fped.2024.1394853

**Published:** 2024-08-05

**Authors:** Fei Qiao, Xinpeng Shang, Fei Jiang

**Affiliations:** Department of Pediatric Orthopaedic, Dalian Women and Children's Medical Group, Dalian, Liaoning, China

**Keywords:** thumb metacarpus, fractures, closed reduction, children, pinning

## Abstract

**Background:**

The treatment of severely displaced Rockwood and Wilkins' type C (RWC) thumb metacarpal basal fractures remains controversial in children. This retrospective study aimed to compare the efficacy of two treatment methods, open vs. closed reduction with pinning of such injuries.

**Methods:**

This study included 30 patients with open physes, 14 boys and 16 girls, who all received either closed or open reduction treatment. The primary outcomes of interest included healing time, complications, and functional results, which were evaluated using the improved Mayo score standard. The minimum follow-up period was 24 months, with a mean of 30.3 months (range 24.0–45.0 months). Statistical significant was defined as *P* < 0.05.

**Results:**

All fractures were healed within 7 weeks after surgery, regardless of which surgical approach was used. However, the recovery time was markedly faster in the closed group, with a mean of 4.2 weeks, than in the open group, with a mean of 4.7 weeks (*P* < 0.05). The operation time for closed group, taking 20 min in average, was also shorter than that for open group (*P* < 0.05). The total incidence of mild complications was lower for patients in the closed group than for patients in the open group (6.3% vs. 21.4%, *P < *0.05). No major complications were observed in either group. In the closed group, a total of 15 patients exhibited excellent outcomes, while only one patient demonstrated good outcomes. On the other hand, in the open group, 12 patients experienced excellent outcomes, whereas two patients had good outcomes. There were no instances of osteomyelitis, refractures or nonunion, avascular necrosis (AVN), or premature physeal closure in either group.

**Conclusion:**

The data from the open group and closed group procedures for severely shifted RWC fractures in children indicate comparable prognoses and complication rates between the two groups. Obviously closed reduction, in particular, offers several advantages over open procedure, including shorter surgical duration, fewer K-wires required, and no need for open incisions. Consequently, closed reduction is the preferred method for treating such RWC fractures.

## Background

Metacarpal fractures of the thumb are uncommon paediatric injuries, accounting for 1%–5% of hand fractures (1–3). Thumb metacarpal base fractures in children can be divided into four types, with Type C being identified as a Salter–Harris (S–H) type II epiphyseal fracture with internal angulation (4). Nonoperative management is typically used for non-displaced (Rockwood and Wilkins' type C) RWC fractures and RWC fractures with an angulation of less than 30° (4–6). However, there remains controversy regarding the management of significantly displaced RWC thumb metacarpal basal fractures. Some experts suggest that successful closed reduction and stable results make the use of a short-arm spica splint or cast immobilization possible (4, 7–9). However, in cases where closed reduction is unsuccessful or irreducible RWC thumb metacarpal base fractures occur, open reduction and internal fixation (ORIF) should be employed (4, 10–12).

Surgical intervention is recommended for RWC fractures angulated >30°, a displacement magnitude of more than two-thirds of the diameter of the growth plate, or a malrotated fracture (6, 8, 9). At our organization, we employ both the closed and open approaches to manage these fractures. The aim of this retrospective study was to compare the imaging and clinical results and the incidence of complications in patients with RWC fractures treated with either method. Our hypothesis posited that closed reduction could be used to effectively manage these fractures with minimal surgical procedures without requiring an incision while maintaining a low incidence of complications.

## Materials and methods

This retrospective study involved 32 patients who sustained severely angulated RWC metacarpal base fractures resulting in acute displacement, excluding open fractures. The study was conducted at a single level of one paediatric trauma centre of Dalian Women and Children's Medical Group from October 2011 to September 2017, following ethical approval from the Institutional Ethical Review Board (approval number DLEY-KY-2021-08), and informed consent was obtained from the patients and their respective parents or custodians. To qualify for inclusion in the study, it was necessary for patients to meet certain specific criteria. These included a fracture angle greater than 30°, a magnitude of fracture displacement exceeding two-thirds of the physeal plate's diameter, or a malformation of angulation as mensurated on either an anterior-posterior oblique radiograph or CT scan. Furthermore, qualifying patients were required to be under 14 years of age with open physes and to have undergone operative procedures and Kirschner wire (K-wire) fixation, performed by two experienced senior surgeons. Patients received treatment through closed reduction and open reduction pinned with K-wires according to two surgeons' habit. Thirty patients were followed up for at least 24 months. Two additional observers retrospectively analyzed the clinical observations and radiographs of the patients, ensuring equally recorded assessments were available for both groups (*p* > 0.05). The modified Mayo score (13) (Table 1) was used to assess the results. Table 2 summarizes the demographics and follow-up findings. The modified Clavien‒Dindo classification was used to classify the complications (14).

**Table 1:** Modified Mayo score^a^.

Category	Points	Examination findings
Pain	25	No pain
	20	Pain only with weather change
	15	Moderate pain on exertion
	15	Slight pain with activities of daily living
	5	Moderate pain with activities of daily living
	0	Pain at rest
Satisfaction	25	Very satisfied
	20	Moderately satisfied
	10	Unsatisfied but fit for work
	0	Unsatisfied and unfit for work
ROM^b^ (IP, MCP, saddle joint)	25	100% of the uninjured thumb
	15	75%–99% of the uninjured thumb
	10	50%–74% of the uninjured thumb
	5	25%–49% of the uninjured thumb
	0	0%–24% of the uninjured thumb
Pulp-to-palm distance^c^	25	100% of the uninjured thumb
	15	75%–99% of the uninjured thumb
	10	50%–74% of the uninjured thumb
	5	25%–49% of the uninjured thumb
	0	0%–24% of the uninjured thumb
Power measurement^d^	25	100% of the uninjured thumb
	15	75%–99% of the uninjured thumb
	10	50%–74% of the uninjured thumb
	5	25%–49% of the uninjured thumb
	0	0%–24% of the uninjured thumb
Sensibility^e^	25	Normal sensibility
	20	Diminished light touch
	15	Diminished protective sensation
	10	Loss of protective sensation
	5	Deep sensation of pressure
	0	Without sensation
Final score(points)	135–150	Excellent
	120–134	Good
	97–119	Fair
	<97	Poor

A score of 97 points or better was considered to be a “satisfactory result”.

^a^ Reference: Parvizi D, Haas FM. Division of Plastic, Aesthetic and Reconstructive Surgery, Department of Surgery, Medical University Hospital of Graz, Austria.

^b^ Rang of movement (sum of IP, MCP, and saddle joint).

^c^ Defined as the distance of the thumb pulp to the metacarpophalangeal furrow of the fifth digit in centimeter.

^d^ Sum of adduction and pinch grip.

^e^ By Semmes-Weinstein monofilaments.

**Table 2:** The demographic characteristics, evaluation results and complications of the patients and fractures.

	CRPP (*n* = 16)	ORIF (*n* = 14)	*p-*value
Age			0.166
Mean age(years)	10.8	9.6	
Sex, *n* (%)			0.984
Female	7	6	
Male	9	8	
Surgery time(min)	20	32	0.000
Complications	1/16	3/14	0.498
Amount of K wire	2	3.7	0.000
Removal of K-wires(weeks)	4.2	4.7	0.047
Mean Angulation(°)			
Preoperation	50.5°	46.2°	0.101
Postoperation	5.0°	4.6°	0.448
The modified Mayo score			0.728
Excllent	15	12	
Good	1	2	
Fair			
Poor			

Statistically significance was set to be *p* < 0.05.

### Surgical technique

The patient lay supine on the operating table, with the afflicted arm abducted. Following the administration of general anaesthesia, the injured arm was prepared and draped before the reduction procedure commenced without tourniquet.

### Closed reduction

**Figure 1:**
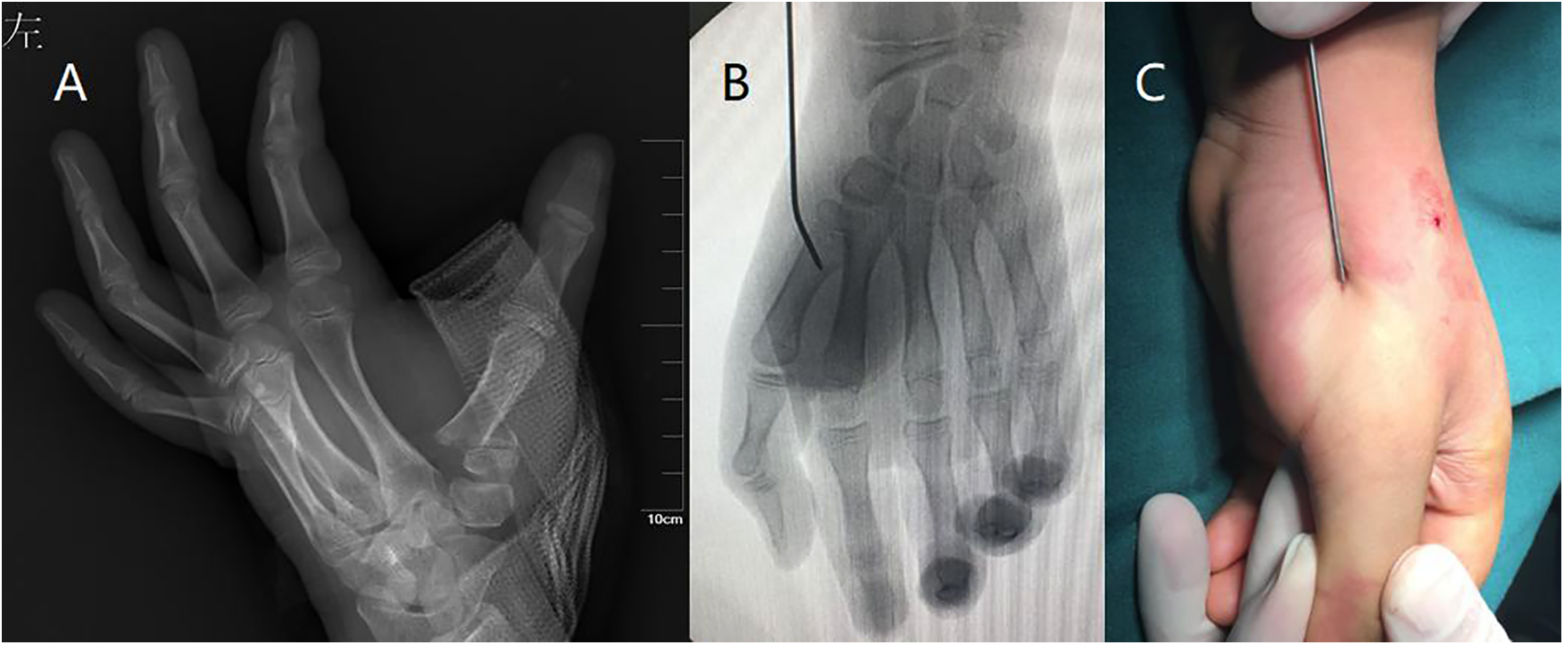
Typical thumb metacarpal base RW-C fracture of 10.5 years old boy. (**A**) AP x-ray of left thumb preoperative. (**B**) Closed reduction of the fracture. (**C**) Aspect of closed reduction.

**Figure 2:**
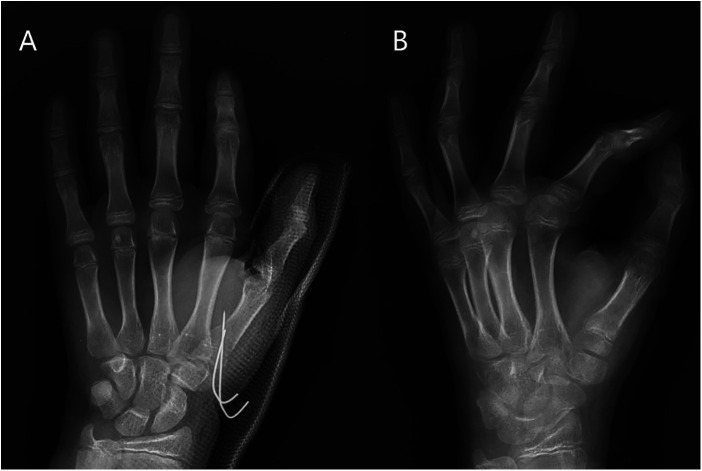
(**A**) Result after closed reduction. (**B**) 8 weeks follow-up x-ray after closed reduction with pinning.

Using a C-arm image enhancer, a lever K-wire 1.5 mm in diameter was meticulously inserted percutaneously into the distal metacarpal fragment while carefully ensuring that it did not surpass the dorsal cortex of the distal fragment. Upon crossing the fracture site, the K-wire was positioned towards the metaphysis to avert physis injury, while additional pressure was applied to the dorsal and medial rims for reduction of the distal block. To maintain anatomic reduction, dual antegrade crossing Kirschner (DACK) wires 1.0 mm in diameter were employed, and their efficacy was confirmed through anteroposterior and lateral radiographs (15) (Figures 1, 2).

### Open reduction

**Figure 3:**
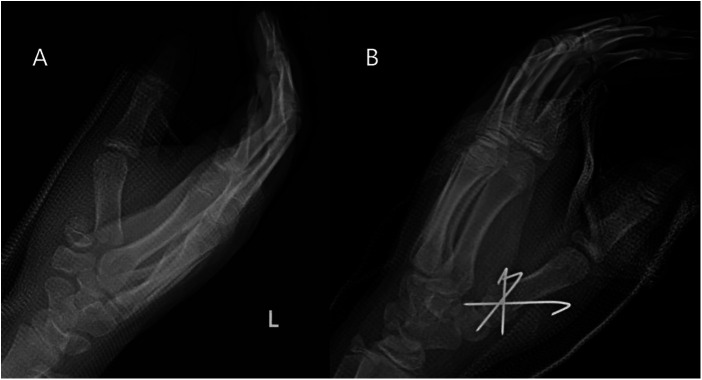
Typical thumb metacarpal base RW-C fracture of 9.3 years old boy. (**A**) AP x-ray of left thumb preoperative. (**B**) Result after open reduction with pinning.

**Figure 4:**
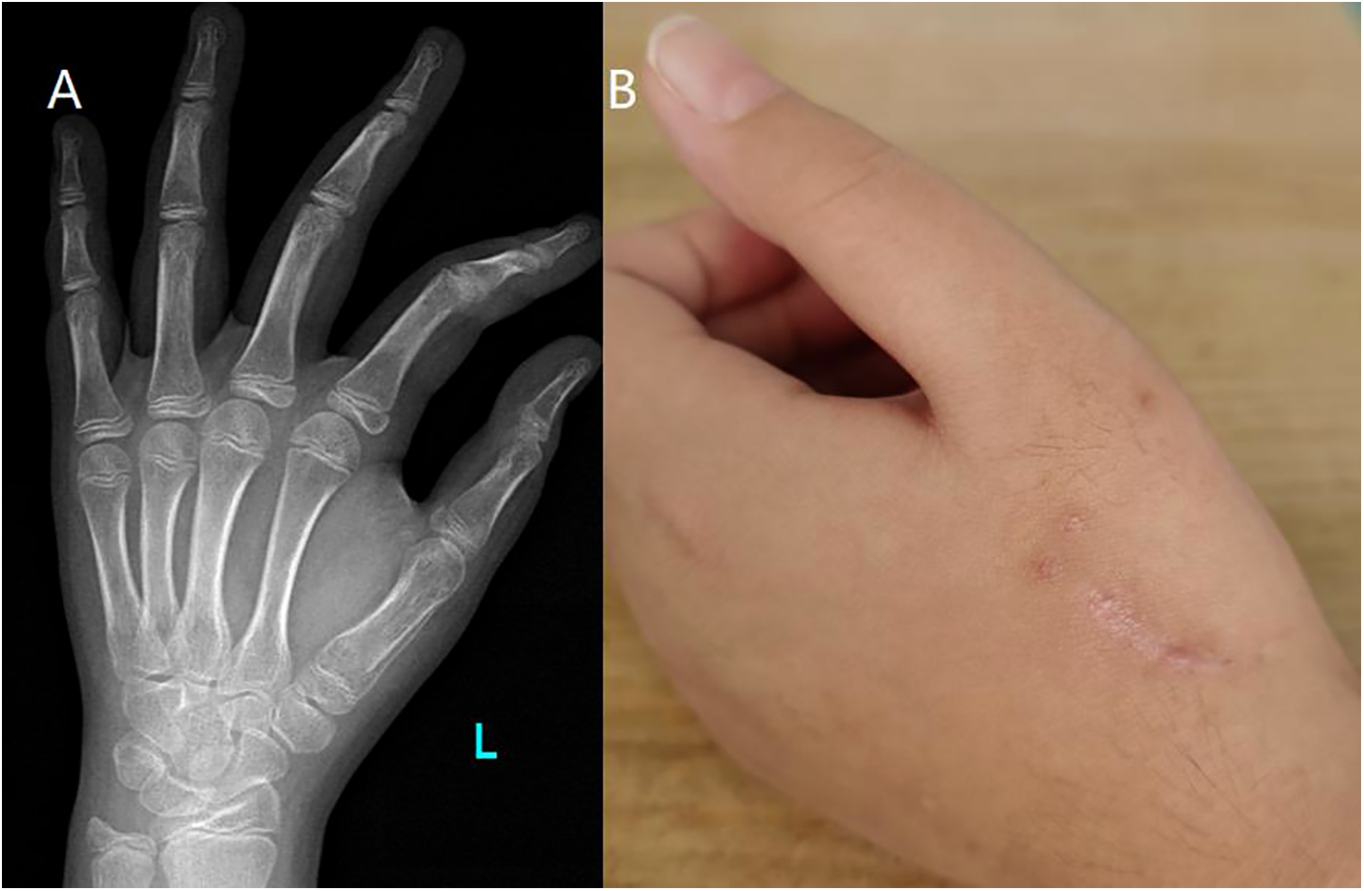
(**A**) 12 weeks follow-up x-ray after open reduction. (**B**) Aspect of open reduction.

After an unsuccessful attempt at manual reduction, a traditional radial approach to the thumb was executed under general anaesthesia. The surgeon was careful to minimize damage to the soft tissue and periosteum. The fracture was then reduced through an open procedure and fixed with 3–4 retrograde crossing Kirschner (RCK) wires 1.0 mm in diameter, which had to transit the epiphysial fragment (4, 10, 11) (Figures 3, 4).

Once the reduction and fixation were successfully completed, the external part of the wire was bent to an angle of 90°. After the operation, the patient's arm was immobilized with a thumb-spica cast that encompassed the entire first ray and was worn for a period of 4–7 weeks. Upon removal of both wires and casting concurrently during outpatient service visits without anaesthesia, the patient was encouraged to perform exercises to help recover the full range of motion (FRM) in the injured thumb.

### Statistical analysis

Statistical analysis was performed using SPSS v22 (IBM Corp., Armonk, NY, USA). The paired-samples *t*-test was employed for normally distributed data, while the independent-samples Mann–Whitney *U*-test was utilized to assess nonnormally distributed data. The level of significance was established at *p* < 0.05.

## Results

The present study involved 30 patients who met the inclusion/exclusion criteria and received surgical intervention. The follow-up period for the patients averaged 30.3 months, ranging from 24.0 to 45.0 months. The closed group consisted of 7 males and 9 females whose ages ranged from 7.5 to 14.0 years, with an average age of 10.8 years. The open group included 6 males and 8 females aged between 6.2 and 12.0 years, with an average age of 9.6 years. There were no significant differences in the demographic data or the distribution of fracture angulation between the two groups (*p* > 0.05). Fourteen patients with displaced thumb metacarpal fractures (average angulation 46.2°, range 35.0°–52.0°) underwent closed reduction (Figure 2) involving fixation with 3/4 retrograde crossing K-wires. Sixteen patients underwent closed reduction with an average angulation of 50.5° (range 40.8°–67.0°), which required fewer K-wires (*p* < 0.05). The operation time for closed group, taking 20 min in average, was also shorter than that for open group (*P* < 0.05). The modified Mayo score criteria were used to assess the clinical results of the two groups. In the closed group, 15 patients had excellent results, and 1 patient had good results, with no poor results reported. In the open group, 12 patients had excellent outcomes, 2 patients had good outcomes, and no poor outcomes were reported. The clinical results between the closed and open groups were not significantly different (*p* > 0.05).

The study revealed that all fractures, regardless of treatment (closed or open), were radiographically healed within 7 weeks, and the average union time was 4.4 weeks (with a range of 4.0–7.0 weeks).There was a significant difference in healing time between the two groups, with closed reduction patients taking 4.2 weeks to heal and open reduction patients taking 4.7 weeks to heal (*P* = 0.047).The total incidence of mild complications was lower for patients in the CRPP group than for patients in the ORIF group (6.3% vs. 21.4%, *P *< 0.05). However, there were no major complications, such as AVN, deep infection, premature physeal closure, or refracture, in either group. In the closed group, 1 mild complication was observed (a pin site infection requiring oral antibiotics), while in the open group, 3 mild complications were observed (3 pin site infections requiring oral antibiotics) (Table 2).

## Discussion

The surgical management of RWC thumb metacarpal base fractures with considerable angulation yielded positive results with a 100% union rate, regardless of whether patients were treated with closed reduction or open reduction. Fortunately, the majority of surgical complications were classified as minor, with an incidence of 6.3% among patients treated with closed reduction and 21.4% among those treated with open reduction; no major complications were identified.

Surgical intervention and fixation of RWC thumb metacarpal base fractures that are displaced with an angulation of more than 30° have been recommended as approaches for maximizing thumb and joint function. Several published manuscripts have addressed surgical treatment for RWC fractures, focusing on techniques utilizing open and closed approaches. The outcomes of these interventions have been positive, with an 80.2%–95.0% success rate reported in the literature. Nevertheless, postoperative complications remain a major challenge, with 8.2%–30.5% of patients experiencing complications, such as pin tract infections, secondary displacement of fracture, premature physis closure, inability to carry out a previous hobby, a loss of grip strength compared with the contralateral side, interference with daily activities (4, 6–12). Our study utilized closed reduction to treat 16 patients with RWC fractures that were angulated at more than 30°. The results were exceptional, with 15 out of 16 patients showing significant improvement in their condition and no reports of major complications. Additionally, we extended the indications for closed reduction to include severely displaced and rotated fractures averaging 48.5° of angulation. The outcomes of these interventions were satisfactory, with no severe complications reported in any patient whose fractures were successfully reduced.

At our medical institution, the two senior paediatric orthopaedic surgeons have different treatment methodologies for managing RWC fractures. While some practitioners prefer open reduction because it directly reduces the fracture and removes any interposed tissue, others favour the use of closed reduction because of its comparatively less invasive nature and potential to mitigate complications, provided that anatomic reduction can be confirmed. Our collected data show that despite negligible differences in clinical findings, closed reduction poses the advantages of a shorter surgery time and the avoidance of incisions. We postulate that despite best efforts to minimize iatrogenic damage at the time of surgery, inadvertent damage to soft tissue, including vessels and tendons, may occur, increasing the likelihood of major complications which did not occur in our series and avoid assuming that could occur theoretically. Consequently, we posit that closed reduction offers a superior alternative to open reduction in terms of reducing the iatrogenic risks involved in the procedure.

Internal fixation is a necessary treatment option for RWC fractures. Jehanno et al. (6) documented a nonoperative treatment method for these fractures, but early secondary displacement occurred in two of the four patients. K-wires can be used to traverse the physis without causing damage. Wiggins proposed the use of a K-wire across the epiphyseal growth plate, which has never been linked to epiphysiodesis (16). The antegrade approach offers several advantages, including ease of fixation, reduced risk of skin infection and K-wire loosening, and fewer K-wires required than retrograde approaches. On average, closed surgery takes 20 min less than open surgery (*P* < 0.05). However, Brüske et al. (17) have shown that intra-articular K-wires can aggravate articular surface lesions, leading to posttraumatic arthritis. As a result, the Iselin technique, which is a percutaneous extra-articular double K-wire intermetacarpal fixation technique, was suggested as an alternative (18).

Niekerk et al. (19) and Greeven et al. (20) reported that the prevalence of complications involving both slight and severe cases treated through the Iselin technique ranged between 33.3% and 50.0% among patients. However, our series of 30 patients revealed no significant complications, apart from pin tract infections in 13.3% of patients. The use of K-wire fixation in our study helped to reduce the probability of secondary movement, superior to the Iselin method.

It is important to note that our study was limited by its retrospective nature and relatively small sample size, which may affect the validity of the results. Additionally, the short-term nature of the study's evaluation is another limitation. As such, further studies with larger sample sizes and longer follow-up periods are required to make definitive conclusions on these two types of surgical procedures.

## Conclusion

In summary, the closed procedure is preferred, but if an open technique is needed, there would be increased risk of minor complications, but both the closed and open reduction with pinning procedures for RWC fractures with an angulation of more than 30° offer satisfactory outcomes with high union rates. Obviously closed reduction, in particular, offers several advantages over open procedure, including shorter surgical duration, fewer K-wires required, and no need for open incisions. Consequently, closed reduction is the preferred method for treating such RWC fractures.

## Data Availability

The original contributions presented in the study are included in the article/Supplementary Material, further inquiries can be directed to the corresponding author. Requests to access the datasets should be directed to Fei Qiao, 229637772@qq.com.
